# Spontaneous pregnancies and determinant factors in infertility: A cross-sectional study

**DOI:** 10.18502/ijrm.v13i10.7775

**Published:** 2020-10-13

**Authors:** Nasrin Saadati, Roshan Nikbakht, Alireza Sattari, Fatemeh Sadat Amininezhad

**Affiliations:** ^1^Department of Community Medicine, School of Medicine, Fertility Infertility and Perinatology Research Center, Ahvaz Jundishapur University of Medical Sciences, Ahvaz, Iran.; ^2^Department of Obstetrics and Gynecology, Fertility Infertility and Perinatology Research Center, Ahvaz Jundishapur University of Medical Sciences, Ahvaz, Iran.; ^3^Fertility Infertility and Perinatology Research Center, Ahvaz Jundishapur University of Medical Sciences, Ahvaz, Iran.

**Keywords:** Male infertility, Female infertility, Spontaneous pregnancy, Epidemiology, Etiology.

## Abstract

**Background:**

The phenomenon of infertility may be derived from different factors - either in males or females or both genders, including few unexplained factors. It is generally managed by medical and surgical treatments.

**Objective:**

To find a relation of occurrence of spontaneous pregnancy (SP) with effective factors in infertility.

**Materials and Methods:**

This cross-sectional study was conducted at two referral infertility centers (university and privacy center) in the southwest of Iran from March 2015 and March 2016 on 655 infertile couples, who were divided in two groups of with (n = 31) and without (n = 624) SP. The variables included female and male age, male smoking, male job, the place of living, the causes of infertility, the type and duration of infertility, and the subgroups of infertility causes.

**Results:**

Infertility may be caused due to both male- and female- related factors (47.5%). While female-related infertility was found in 31.5%, male-related infertility in 14.5%, and infertility due to unexplained factors in 6.6% of our patients. The rate of SP was 4.7%, which had a significant relation with the duration of infertility (p = 0.01), with women's age (p = 0.048), unexplained infertility (p = 0.001), and husband's job (p = 0.004).

**Conclusion:**

The occurrence of SP in infertile couples was related to age of the female partner, the duration of unexplained infertility, and the male partner's job.

## 1. Introduction

Today, infertility is considered as one of the most pertinent public health concerns in all human societies. Based on recent studies by WHO, approximately 8-10% of couples somehow face fertility-related problems, which means that about 50-70 million couples are facing this problem worldwide (1, 2). Several factors can cause infertility in couples including biological, physiological, environmental, or acquired factors. The infertility treatment is generally based on the cause of infertility; however, some infertile couples do not follow-up their treatment properly (3). A follow-up of Iranian and Turkish infertile patients after an ART failure showed that Iranian patients ceased the treatment more than the Turkish. The reasons reported were economic problem, hopelessness, distress of drug side effect, achieving pregnancy, child adoption, lack of spouse partnership and divorce. Nevertheless, the rate of spontaneous pregnancy (SP) was significantly higher in Iran than in Turkey. There was a correlation between the duration of infertility and female factor infertility with SP (4).

The aim of this study was therefore to find a correlation of SP determinant factors in infertility in Ahvaz, Iran.

## 2. Materials and Methods

This cross-sectional study was conducted on 655 couples reffered to two referral infertility centers (210 coupls from Imam Khomeini University Hospital and 445 couples from Zakeria Infertility Center) in Ahvaz, Iran between Mrach 2015-March 2016. Inclusion criteria were all of couples came to the two referral center for treatment of infertility at that time. Exclusion criteria included couples with male-related infertility with azoospermia and the patients who could not be followed-up after their visit to the infertility centers were excluded from the study.

Moreover, the patients' information regarding the variables such as female and male age, duration and type of infertility, number of marriage for both women and men, jobs and status of smoking for male, place of living, causes of infertility -including the female-related (e.g., ovulation factor, tubal factor, uterine factor, endometriosis), male-related, male- and female-related factors, and unknown infertility factors- and the occurrence of SP after ceased treatment or no treatment at that time were extracted from their records. The infertility causes in males were based on abnormal semen parameters according to the WHO 2010 criteria (5). SP was defined as a positive pregnancy test with a following an occurrence of pregnancy without any medical or surgical intervention in female, this included the occurrence of pregnancy three months after a medical therapy, six months after hysterosalpingography, six months after saline infusion sonohysterography, office or surgical hysteroscopy, or diagnostic or surgery laparoscopy or tuboplasty and an occurrence of pregnancy without any intervention in male, which consisted of an occurrence of pregnancy 12 months after varicocelectomy or use of any effective drugs on spermatogenesis (6).

### Ethical consideration

This study was approved by the Ethics Committee of Ahvaz Jundishapur University of Medical Sciences. Informed consent was obtained from all couples for the use of their data.

### Statistical analysis 

Data were statistically analyzed using the SPSS version 18 (Statistical Package for the Social Sciences, SPSS Inc., Chicago, IL, USA). Additionally, *t* test and Chi-square test were respectively used to compare the quantitative and qualitative variables between groups. The conventional p-value of ≤ 0.05 was considered as an overall significant level.

## 3. Results

Six hundred fifty-five infertile couples participated in the study. The SP occurred in 31 (4.7%) during the study period (Table I). Frequency of the causes of infertility was defined in Table I.

**Figure 1:**
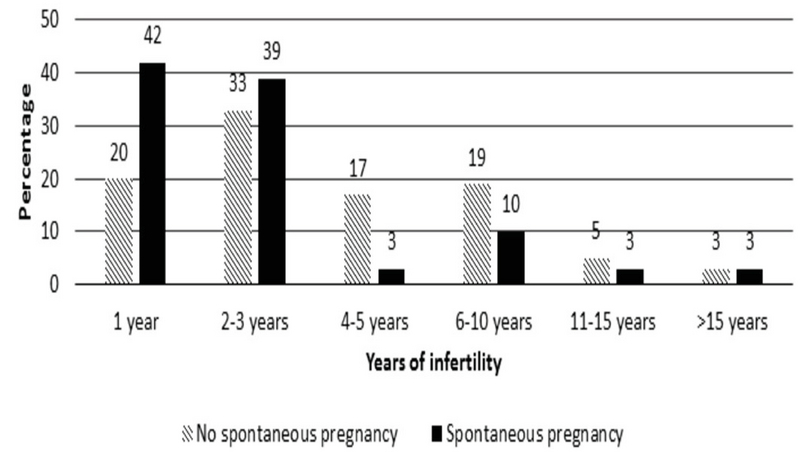
Duration of infertility.

The rates of subgroups of female causes of infertility included ovulatory factor in 164 (31.72%) cases, tubular factor in 99 (19.14%), uterine factor in 50 (9.67%), endometriosis in 4 (0.77%), and at least two of these factors in 200 (38.68%) cases. The high frequent subgroup belonged to the couples with a 2-3 yr of infertility, followed by 1 yr, and then 6-10 yr in two groups (Figure 1).

The mean female age was significantly lower in women with SP (28.17 ± 6.2 yr) than those without SP (30.64 ± 6.29 yr) (p = 0.048). The rate of SP among cases with a duration of infertility ≤ 3 yr and > 3 yr were 80.1% and 54%, respectively (p = 0.01). No statistically significant relationship was observed between primary or secondary infertility with SP (p = 0.46). The rate of frequency of SP based on combined, unexplained, female, and male infertility causes were 45.2%, 25.8%, 19.4%, and 9.7%, respectively. Interestingly, however, there was a statistically significant relationship between unexplained infertility and SP (p = 0.001). Table I. It was found that 64.5% of the cases with SP were < 30 year old, 29% were 30-35 years old, while 6.5% were > 35 year. Further, no significant correlation between SP and age groups were observed (p = 0.81). Also, SP was significantly related to the kind of male's job (p = 0.004)

**Table 1:** Characteristics of 624 infertile couples without spontaneous pregnancy and 31 infertile couples with spontaneous pregnancy


**Variable**		**Total infertile couples (n = 624)**	**Infertile couples with spontaneous pregnancy (n= 31)**	**P-value${}^{a}$**
**Age (yr)**				
	**Female**	30.49 ± 6.3	28.17 ± 6.2	0.048
	**Male**	34.85 ± 7.4	31.87 ± 6.4	0.056
**Female age group**				
	**< 30 yr**	301 (48.2)	20 (64.5)	0.081
	**30-35 yr**	174 (27.8)	9 (29)	
	**> 35 yr**	149 (23.8)	2 (6.5)	
**Male age group**				
	**≤ 50 yr**	594 (95.2)	31 (100)	0.3
	**> 50 yr**	30 (4.8)	0 (0)	
**Male smoking**		118 (18.9)	6 (19.4)	0.9
**Male job**				
	**Catering**	285 (45.7)	10 (32.3)	0.004
	**Professional**	155 (24.8)	5 (16.1)	
	**Government personnel**	137 (22)	12 (39)	
	**Military**	26 (4.2)	3 (9.6)	
	**Farmer**	10 (1.6)	0 (0)	
	**Workless**	1 (0.1)	1 (3)	
	**Missing**	10 (1.6)	0 (0)	
**Place of living **				
	**Urban**	549 (88)	27 (87.1)	0.79
	**Rural**	52 (8.3)	3 (9.7)	
	**Missing**	23 (3.7)	1 (3.2)	
**Duration of infertility**				
	**Mean ± SD (yr)**	4.2 ± 4.5	3.03 ± 3.2	0.15
	**Infertility ≤ 3 yr**	337 (54)	25 (80.1)	0.01
	**Infertility > 3 yr**	275 (44.1)	6 (19.9)	
	**Missing**	12 (2)	0	
**Type of infertility**				
	**Primary **	389 (62.3)	17 (54.8)	0.46
	**Secondary**	235 (37.6)	14 (45.1)	
**Causes of infertility**				
	**Combine **	297 (47.6)	14 (45.2)	0.69
	**Female**	198 (31.7)	8 (25.8)	0.91
	**unexplained**	37 (5.6)	6 (19.4)	0.001
	**Male **	92 (14.7)	3 (9.7)	0.50
${}^{a}$ The student *t* test was used for qualitative variables and the Chi-square test for quantitative variables. The values are presented as Mean ± SD or N (Percentage). The p-value < 0.05 was considered as significant				

## 4. Discussion

In the study, the rate of SP was reported to be 4.7%. Frequency the occurrence of SP in order to the causes of infertile subgroups was combined, unexplained, female and male factor, respectively. So, it showed the rate of SP in subgroup unexplained infertility was more than the other subgroups. There was no statistically significant relationship between primary and secondary infertility with SP, however, the duration of infertility for ≤ 3 years was associated with a significant increase in the rate of SP. Also, the mean female age was significantly lower in women with SP. The type of male's job was also significantly related to the SP.

The limitation of diagnostic procedure for predicting pregnancy is well-known, for example, a study exploring pregnancy rate after hystersalpingo-contrast sonography showed that when two tubes were closed, the pregnancy rate was reduced to 15.04%, so the occlusion of both the tubes have a false positive result. In this study, a univariate analysis showed that younger age was positively related to initiation of pregnancy as found in our study; however, the infertility type, duration of menstrual cycle, dysmenorrhea, and parity were unrelated to pregnancy (7). Another study showed that some patients with premature ovarian insufficiency have an intermittently ovarian activity with an overall 5% probability of pregnancy (8). In addition, we know that the current WHO criteria for semen quality do not distinguish between fertile and sub-fertile (9). So, we could expect an occurrence of SP despite the results of para-clinic.

Further, a study was conducted to determine the reasons and incidence for withdrawal from a waiting list for IVF. It was reported that about 13% of the patients gave up before going through their first IVF cycle and 37% of them has an SP, which is the same rate of SP as reported in our study. Most of the pregnancies occurred within three months after the patient had been set up for a waitlist, which suggests that psychological factors might be effective (10). As in our study, they showed that we should give hope, especially to young couples with unexplained infertility, although they might have to wait for a long time. They also reported that SPs mostly occurred in 2-4 years of successful or unsuccessful IVF attempts (11). Similar to our findings, another study determined that the younger women and also shorter duration of infertility had higher rate of spontaneous pregnancy after surgery (12). Another study showed that SP occurred after the management of autoimmune disorders (13).

Moreover, the initial experimental management for six-month results were reported in couples with unexplained sub-fertility and that an intermediate prognosis of natural conception is effective (3, 14). We also know that female age has a prominent negative impact on fertility and the solutions are not simple, however, an alternative like validated dynamic models could predict both natural and ART-mediated in short time (15). Another study showed that allostatic load that is specific in chronic situation like infertility was not associated with fertility outcomes like conception, spontaneous abortion, and live birth (16). However, a cohort study on 4,999 couples showed that the prediction ranges could help in counselling couples for at least two years after their fertility work-up (17). Additionally, a longitudinal cohort study showed a 71.6% live birth after ART and 28% after SPs or both up to five years (18). The small rate of SP in our study might have been affected by the environment, especially heat intensity, genetics, socioeconomic factors, nutrition, and obesity. On the other hand, many studies have been conducted for increasing fertility, for example, in a randomized controlled trial, the effect of endometrial scratching was conducted in couples with unexplained infertility and a good prognosis, and we must wait for their results (19).

In addition, several articles have pointed the lifestyle factors such as diet, use of fish consumption, exercise, intervention for losing weight, and optimized counseling associated with the SP and IVF success rate (20). Obesity affects SP in sub-fertile ovulatory women, so it needs to be managed (9). While, the limitations of this study were its retrospective pattern and short follow-up, the strength of the study was the evaluations of SP infertile people from a specific geographical region and the number of participants in the study; therefore, we suggest a multi-centric research. In addition, we can expect to achieve an SP despite the results of diagnostic tests and failures of the treatment especially in the unexplained infertility. The occurrence of SP in infertile couples relate to the age of the female partner, the duration of infertility, unexplained factors, and also the job of the male partners.

##  Conflict of Interest 

The authors declare that there is no conflict of interest.
